# Intensive insulin therapy versus plasmapheresis in the management of hypertriglyceridemia-induced acute pancreatitis (Bi-TPAI trial): study protocol for a randomized controlled trial

**DOI:** 10.1186/s13063-019-3498-x

**Published:** 2019-06-18

**Authors:** Xiao Song, Di Shi, Qinghong Cui, Shanshan Yu, Jing Yang, Priscilla Song, Joseph Walline, Jun Xu, Huadong Zhu, Xuezhong Yu

**Affiliations:** 1 Department of Emergency, Peking Union Medical College Hospital, Chinese Academy of Medical Sciences & Peking Union Medical College, Beijing, China; 2 Center for the Humanities and Medicine, The University of Hong Kong, B926, 9F, Run Run Shaw Tower, Centennial Campus, Pokfulam Road, Hong Kong, China; 3 Accident and Emergency Medicine Academic Unit, 2/F, Main Clinical Block and Trauma Centre, Prince of Wales Hospital, The Chinese University of Hong Kong, Shatin, NT Hong Kong SAR

**Keywords:** Hypertriglyceridemia-induced acute pancreatitis, Insulin, Plasmapheresis, Triglyceride-lowering

## Abstract

**Background:**

It is widely agreed that triglyceride (TG)-lowering therapy is imperative in early hypertriglyceridemia-induced acute pancreatitis (HTG-AP). Intravenous insulin with or without heparin, and plasmapheresis are available regimens. However, there is no consensus on first-line therapy.

**Methods/design:**

The Bi-TPAI trial is a multicenter, parallel group, randomized, controlled, non-inferiority trial in patients with early HTG-AP. The Bi-TPAI trial will include 220 patients with HTG-AP from 17 large tertiary hospitals in China. Patients assigned to the intensive insulin group will be administered an intravenous continuous infusion of regular human insulin at a rate of 0.1 units/kg·h and up to 0.3 units/kg·h. Patients allocated to the plasmapheresis group will receive standard-volume plasmapheresis. The primary endpoint is the time it takes for the TG level to reduce to 500 mg/dl. The secondary endpoints are ICU and hospital lengths of stay, 28-day mortality, severity of HTG-AP, incidence of hypoglycemia, HTG-AP complications, and cost-effectiveness.

**Discussion:**

The Bi-TPAI trial will prove that intensive insulin therapy is non-inferior to plasmapheresis. Intensive insulin therapy should be an effective, safe, available, and cheaper triglyceride-lowering therapy for hypertriglyceridemia-induced acute pancreatitis.

**Trial registration:**

ClinicalTrials.gov, NCT03342807. Registered on 5 Nov 2017.

**Electronic supplementary material:**

The online version of this article (10.1186/s13063-019-3498-x) contains supplementary material, which is available to authorized users.

## Background

Hypertriglyceridemia-induced acute pancreatitis (HTG-AP) occurs in the presence of severe hypertriglyceridemia (sHTG) (triglyceride (TG) > 1000–2000 mg/dl) in the absence of other causes [1]. The manifestation of HTG-AP is similar to that of acute pancreatitis (AP) due to other causes. It is usually treated with conservative management and TG-lowering therapy to prevent recurrence of pancreatitis and systemic inflammatory responses, targeting a serum TG level < 500mg/dl [2].

Current guidelines have not yet defined first-line TG-lowering therapies. Previous studies investigating the treatment of HTG-AP with insulin alone, insulin in combination with heparin, plasmapheresis or hemofiltration techniques, and/or fibrates included only case reports and small case series [3–6]. Plasmapheresis can reduce serum TG levels effectively and quickly, but raises concerns about its cost-effectiveness, complications, and staffing resources. Insulin deceases TG by promoting synthesis and activity of lipoprotein lipase (LPL), which hydrolyzes TG into fatty acids and glycerol and facilitates storage of the fatty acids in adipocytes [7]. Enthusiasm for heparin has been waning due to a transient rise in LPL followed by increased degradation and depletion of circulatory LPL [8, 9].

There have been reports where insulin was the main method used for reducing TG levels. However, using an insulin infusion as a therapeutic strategy for lowering TG levels has not yet been adequately studied. No studies have been conducted to identify the better route of insulin delivery (either intravenous or subcutaneous) to treat HTG-AP, although insulin has been used as a continuous infusion in almost all case series and reports. There is also no consensus on the dose of insulin or appropriate ending time. There have been case series or trials targeting euglycemia instead of serum TG level. Diverse insulin strategies yield inconsistent TG-lowering rates. The effectiveness of insulin is underestimated in some studies. We propose using an “intensive insulin therapy in HTG-AP”, which means a continuous insulin infusion until serum TG is < 500 mg/dl, even if the blood glucose level is acceptable.

The Bi-TPAI trial is designed to investigate whether intensive insulin therapy is as effective as plasmapheresis in lowering TG in patients with HTG-AP.

## Methods

### Trial design and setting

**Fig. 1:**
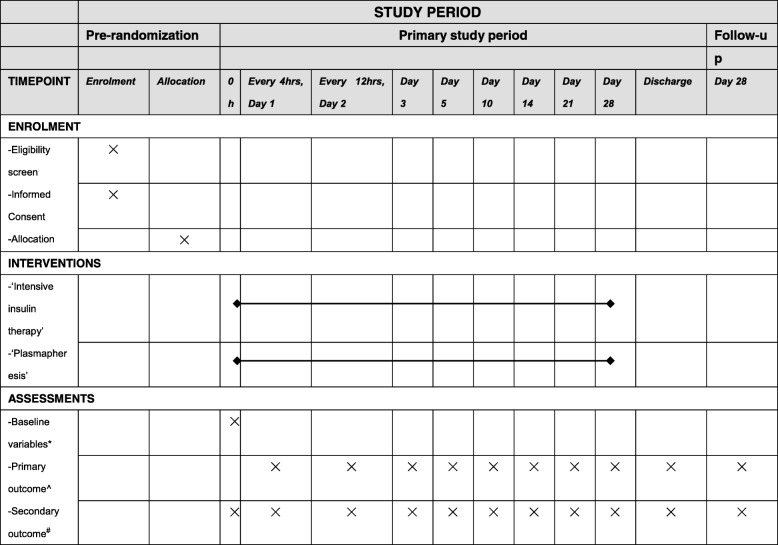
Standard Protocol Items: Recommendation for Interventional Trials (SPIRIT) schedule of enrolment, intervention and assessments. *Baseline variables: age, gender, body mass index (BMI), diet, history of alcoholism, comorbidity, family history of hypertriglyceridemia or hypertriglyceridemia-induced acute pancreatitis (HTG-AP). ^Primary outcome: triglyceride (TG) level < 500 mg/dl. ^#^Secondary outcome: ICU- and hospital length of stay (LOS), 28-day mortality, HTG-AP severity scores, incidence of hypoglycemia, HTG-AP complications, and cost-effectiveness

The Bi-TPAI trial is a national, multicenter, parallel group, open, non-inferiority, randomized controlled trial that will include 220 patients with HTG-AP, from 17 large tertiary hospitals in China. The Bi-TPAI trial will be conducted according to the principles of the Declaration of Helsinki [10]. The Institutional Review Board of Peking Union Medical College Hospital, Chinese Academy of Medical Sciences & Peking Union Medical College in Beijing, China, approved the trial protocol (version 2.0, reference number ZS-1340, date 20 June 2017). The schedule of enrolment, intervention and assessments following the Standard Protocol Items: Recommendation for Interventional Trials (SPIRIT) guidelines (Additional file 1) is presented in Fig. 1.

### Population

Inclusion criteria are patient age 18–80 years, diagnosis of AP, and TG level > 1000 mg/dl. Exclusion criteria are as follows: (1) other etiology of AP; (2) allergies to plasma, human albumin, and heparin; (3) contraindications for plasmapheresis: mental disorders, circulatory failure, unstable heart or cerebral infarction, intracranial hemorrhage, or severe brain edema; (4) more than 72 h since onset of AP.

There is a research coordinator at each hospital to promote and coordinate the trial. Patients will be informed of the purpose and procedures involved, and the potential risks and benefits of the study. The participants’ written consent will be obtained. Participants will be allowed to withdraw from the trial at any time without consequence.

### Treatment arms and co-interventions

Participants will be allocated to either the intensive insulin group or the plasmapheresis group. The intervention will be started within 24 h after diagnosis in all cases. The intensive insulin group will be administered a continuous intravenous infusion of regular human insulin at a rate of 0.1 units/kg·h and up to 0.3 units/kg·h, titrated to maintain TG < 5.6 mmol/l (500 mg/dl): 5% or higher concentration of intravenous dextrose will be continuously given simultaneously according to fluid and calorie demands. Dextrose infusion is titrated to avoid hypoglycemia and maintain blood glucose levels between 120 and 200 mg/dl (6.7–11.1 mmol/l) during treatment. The plasmapheresis group will receive standard-volume plasmapheresis (1–1.5 plasma volume) every 24 h until the TG level is < 5.6 mmol/l. The plasma volume is calculated as follows:

$$ \mathrm{Plasma}\ \mathrm{volume}=0.07\times \left(\mathrm{body}\ \mathrm{weight}\ \mathrm{in}\ \mathrm{kilograms}\right)\times \left(1-\mathrm{HCT}\right) $$

General treatment measures include pancreatic rest (limited oral intake), aggressive intravenous hydration, and analgesia. Patients in the plasmapheresis group who have diabetes mellitus will receive intravenous insulin drips. This insulin will be added in a common ratio with dextrose (generally 1:4~6), aiming to control the patient’s diabetes mellitus with minimal insulin, and not to reduce serum TG.

### Study endpoints

The primary endpoint is the time it takes for tTG to drop to 500 mg/dl. The secondary endpoints are (1) ICU and hospital length of stay (LOS); (2) 28-day mortality; (3) severity of HTG-AP - this will be reported according to the revised Atlanta criteria [11], the Acute Physiology and Chronic Health Evaluation II (APACHE II) score, the Ranson score and computed tomography (CT) grading; (4) incidence of hypoglycemia; (5) HTG-AP complications according to the revised Atlanta criteria [11]; and (8) cost-effectiveness.

### Sample size calculation

The Bi-TPAI trial is a non-inferiority randomized controlled trial. Respective studies and series reported 60–72% of cases with TG < 5.6 mmol/l after one or two sessions of plasmapheresis [4, 12]. A total of 176 participants (88 per group) is required to show non-inferiority of intensive insulin therapy compared to plasmapheresis therapy, with a two-sided significance level of 5% and statistical power of 80%. To account for drop-out and missing data, the sample size is increased by 20% to a final sample size of 220 participates (110 per group).

### Randomization and blinding

Stratified block randomization will be performed centrally in a 1:1 ratio using SAS software (SAS Institute, Inc., Cary, NC, USA). Within each stratum, a random block size of 4 will be used. After written informed consent is obtained, the participant is assigned to one of the two treatment arms. Due to the nature of the investigational treatment, participants and researchers are not blinded to treatment allocation. All analyses will be performed in a blinded fashion.

### Data management

Clinical data will be collected locally via Research Electronic Data Capture (REDCap), an Internet-based electronic case report form (CRF). The research coordinator at each hospital will provide training in study procedures to improve adherence to the protocol. Furthermore, they will check CRFs and contact responsible staff members to ensure data quality.

### Statistical methods

The primary endpoint will be analyzed based on an intention-to-treat principle, regardless of whether they completed the originally allocated treatment study protocol. Any reasons for protocol violations will be described. All *p* values are two-tailed, and the significance level will be a *p* value <0.05.

Continuous variables will be expressed as means and standard deviations (normal distribution) or median with interquartile range (skewed distribution). Student’s *t* test (normal distribution) or Mann-Whitney *U* test (skewed distribution) will be used for group comparisons. Data will be presented as frequencies and percentages for categorical variables. Categorical variables will be compared using Pearson’s chi-squared test or Fisher’s exact test as appropriate. Time-dependent data will be presented as Kaplan-Meier curves. Statistical uncertainty will be expressed in terms of a relative risk and 95% confidence intervals.

Data will be analyzed using the statistical program SPSS version 20.0 for Windows (SPSS Inc., Chicago, IL, USA).

## Discussion

As hypertriglyceridemia may cause more severe acute pancreatitis and worse symptoms, it is imperative to resolve the underlying etiology. There are many available therapies for hypertriglyceridemia-induced acute pancreatitis, including oral lipid-lowering agents, intravenous insulin with or without heparin, and plasmapheresis [13]. Effectiveness, side effects, cost, and availability may influence the final choice of therapy.

Several studies have reported on the effectiveness and safety of insulin [14, 15]. A retrospective cohort study in 2016 showed a significant TG decrease at each time interval in patients treated with intravenous insulin and plasmapheresis, but no difference in clearance rate or length of stay between intravenous insulin and plasmapheresis [16].

A prospective randomized controlled trial (RCT) showed the superiority of high-volume hemofiltration (HVHF) over insulin combined with heparin in lowering TG levels. However, as He, et al. demonstrated in 2016, early HVHF cannot improve clinical outcomes [17]. As for heparin, the effects of heparin on triglycerides are transient because of increased transport of LPL to the liver for degradation [18].

Plasmapheresis is relatively expensive and labor intensive and is associated with complications. Intensive insulin therapy costs much less than plasmapheresis. Researchers calculated that the direct cost to a hospital would be around US$36, if a patient requires 120 units of regular insulin within 24 h. However, a 24-h plasmapheresis course would cost US$3800 plus the variable cost of associated blood products. There is at least 100-fold cost difference between the two therapies [16].

At present, no studies have been conducted to identify the best route (intravenous or subcutaneous) for insulin treatment or the best dose to administer insulin in order to treat HTG-AP. The Bi-TPAI trial plans to include 220 patients from multiple hospitals and should provide adequate power to answer the question of whether intensive intravenous insulin therapy significantly lowers serum TG similarly to plasmapheresis.

One important limitation of the Bi-TPAI trial is that blinding is not possible due to the nature of the intervention. However, attending physicians and nurses who have no specific interest in the trial will be included. Statistical analysis will be performed in a blinded fashion. We hope to answer the question of whether intensive insulin can effectively reduce serum TG and whether this approach is safe and cost-effective.

### Trial status

Bi-TPAI trial started recruiting patients on 13 June 2018. Protocol version 2.0 was applied (20 June 2017). The recruitment is expected to be completed before 2021.

## Additional file

Additional file 1: SPIRIT checklist. (DOCX 32 kb)

## Data Availability

The datasets generated and/or analyzed during the current study are available from the principal investigator (Xiao Song and Jun Xu) on reasonable request.

## Abbreviations

AP: Acute pancreatitis
APACHE II: Acute Physiology and Chronic Health Evaluation II
BMI: Body mass index
CRF: Case report form
HTG-AP: Hypertriglyceridemia-induced acute pancreatitis
HVHF: High-volume hemofiltration
LOS: ICU and hospital length of stay
LPL: Lipoprotein lipase
RCT: Randomized controlled trial
REDCap: Research Electronic Data Capture
sHTG: Severe hypertriglyceridemia
TG: Triglyceride
